# Imaging features in incident radiographic patellofemoral osteoarthritis: the Beijing Shunyi osteoarthritis (BJS) study

**DOI:** 10.1186/s12891-019-2730-x

**Published:** 2019-08-07

**Authors:** Yudian Qiu, Chutong Lin, Qiang Liu, Qunjie Zhong, Ke Tao, Dan Xing, Hu Li, Jianhao Lin

**Affiliations:** 1 Institution of Arthritis, Peking University People’s Hospital, NO11 South street of Xizhimen, Xicheng district, Beijing, China; 2 Department of Radiology and Biomedical Imaging, University of California, San Francisco, San Francisco, CA USA

**Keywords:** Patellofemoral osteoarthritis, Osteophyte, Joint space narrowing, Radiograph

## Abstract

**Background:**

The present study aims to describe the imaging features in incident radiographic patellofemoral osteoarthritis (RPFOA) population in a Chinese suburban area.

**Methods:**

The Beijing Shunyi osteoarthritis (BJS) study was a population-based, longitudinal and prospective study. Residents were recruited by randomized cluster sampling in 2014 and were followed 3 years later. Home interviews and clinical examinations were performed; weight-bearing posterior-anterior semi-flexed (45-degree) views of the tibiofemoral (TF) joints and skyline (45-degree) views of the patellofemoral (PF) joints were included. For each batch of study films (*n* = 100), 20 films from the year 2014 and 20 previously read PF radiographs were fed back to test inter−/intra-reader repeatability. The imaging features of incident RPFOA were analyzed. Narrative statistics, independent-sample t-tests, and nonparametric tests were performed.

**Results:**

A total of 1295 participants (2590 knees) were recruited at baseline in 2014, and 967 (74.7%) residents were followed in 2017. Of all the knees (*n* = 1537) without RPFOA at baseline, 139 knees (13.3%) across 119 people developed incident RPFOA. Compared with the whole population, age (*p* = 0.031), body mass index (BMI, *p* = 0.042), and incidence of knee pain symptoms (*p* < 0.01) were significantly different in the incident RPFOA population, while range of motion (ROM, *p* = 0.052) and gender (0/1, *p* = 0.203) showed no significance. In the incident population, the changes of each imaging indicator grade were evaluated—lateral patellofemoral osteophyte (LPOST, increased by 1.02), medial patellofemoral osteophyte (MPOST, increased by 0.49), lateral joint space narrowing (LJSN, increased by 0.30), medial joint space narrowing (MJSN, increased by 0.06); indicator grade progress decreases, respectively. The progress of LPOST was the fastest among the four indicators (*p* < 0.01).

**Conclusions:**

In this population-based longitudinal study, among the incident RPFOA population, the imaging indicators show that marginal patellofemoral osteophyte is more pronounced than patellofemoral joint space narrowing. LPOST is the fastest-progressing indicator among all the radiographic features, which is also the most common imaging manifestation of RPFOA. In the incident RPFOA population, the proportion of elders, women, higher-BMI individuals, and people suffering knee pain is more than the normal population.

## Background

Knee osteoarthritis (OA) is the most common joint disorders. It is a leading cause of pain and disability among adults [1, 2], impacting many health outcomes and leading to a high burden among the Chinese population [3]. Although osteoarthritis prevalence and incidence estimates have varied somewhat across studies [1, 4], there is agreement that a substantial proportion of adults are affected. The National Health Interview Survey estimated that 14 million people in the US have symptomatic knee OA [5].

Among knee OA patients, patellofemoral osteoarthritis (PFOA) is relatively common, which is one of the main causes of knee pain. Most studies investigating symptoms and disability in knee OA have defined OA as a disease in the tibiofemoral compartment. However, the disease is not limited to this compartment and may occur in the patellofemoral (PF) compartment, separately or in conjunction with the tibiofemoral compartment. Many factors can lead to osteophyte formation, cartilage damage, subchondral sclerosis, and PF joint subluxation. Female patients are more common in the PFOA population [6]. Previous literature had reported imaging evidence of PFOA in 17.1–34% of women and 18.5–19% of men [7].

Since there are many definitions and classifications of radiographic patellofemoral osteoarthritis (RPFOA), no internationally recognized quantitative diagnostic criteria have been made. We defined a subject as having RPFOA on skyline view when (1) the osteophyte score was ≥2; (2) the joint space narrowing (JSN) score was ≥2 and the osteophyte score was ≥1; (3) the JSN score was 3, which refers to Felson and Baker et al. [8, 9], according to the International Association of Osteoarthritis (OARSI) atlas [10, 11] classification (grades 0–3; 0 = normal, 1 = mild, 2 = moderate, 3 = severe), RPFOA can be diagnosed if any criteria is met. In this study, the imaging features of incident RPFOA patients were analyzed from the aspects of PF joint space narrowing (medial / lateral) and PF joint osteophyte (medial / lateral).

## Methods

The Beijing Shunyi osteoarthritis (BJS) study was a population-based, longitudinal, and prospective study of knee osteoarthritis. The BJS study was approved by the Ethics Committee of Peking University People’s Hospital. Every resident who participated in the survey had signed the informed consent document. This study was based on a random cluster sampling method in 2014. Fourteen villages in Shunyi District, Beijing, China were selected. The inclusion criteria for this study were: (1) residence in Shunyi District, Beijing, and (2) age over 50 years. Exclusion criteria: (1) patients with rheumatoid arthritis, (2) physically disability, (3) mental retardation, (4) patients with advanced malignant tumors or bedridden patients, and (5) people recently living or working outside for more than 6 months. And the rejection criteria: (1) people who did not meet the inclusion criteria, (2) people who met the exclusion criteria, (3) people who failed to complete the main records, and (4) people who showed obvious incompatibility in the survey. Respondents who met any of the criteria mentioned above were withdrawn from the survey.

### Clinical data collection

A total of 1295 residents aged over 50 were enrolled in the baseline and were followed up the same month in 2017 as the BJS study. The baseline and follow-up surveys were standardized questionnaires completed at home or in the survey center. Imaging features (weight-bearing posterior-anterior semi-flexed [45-degree] view of radiographs at TF joints and skyline [45-degree] view of radiographs at PF joints) were performed in local community hospitals. Clinical examinations including height, weight, range of motion (ROM), chair stand test, and 50-ft walk test were performed by the same two clinicians in both 2014 and 2017. In this study, gender, age, height, body mass index (BMI), and knee joint ROM were selected, combined with the results of PF axial film, to summarize the distribution and characteristics of the incident RPFOA population.

### Imaging data analysis

**Fig. 1:**
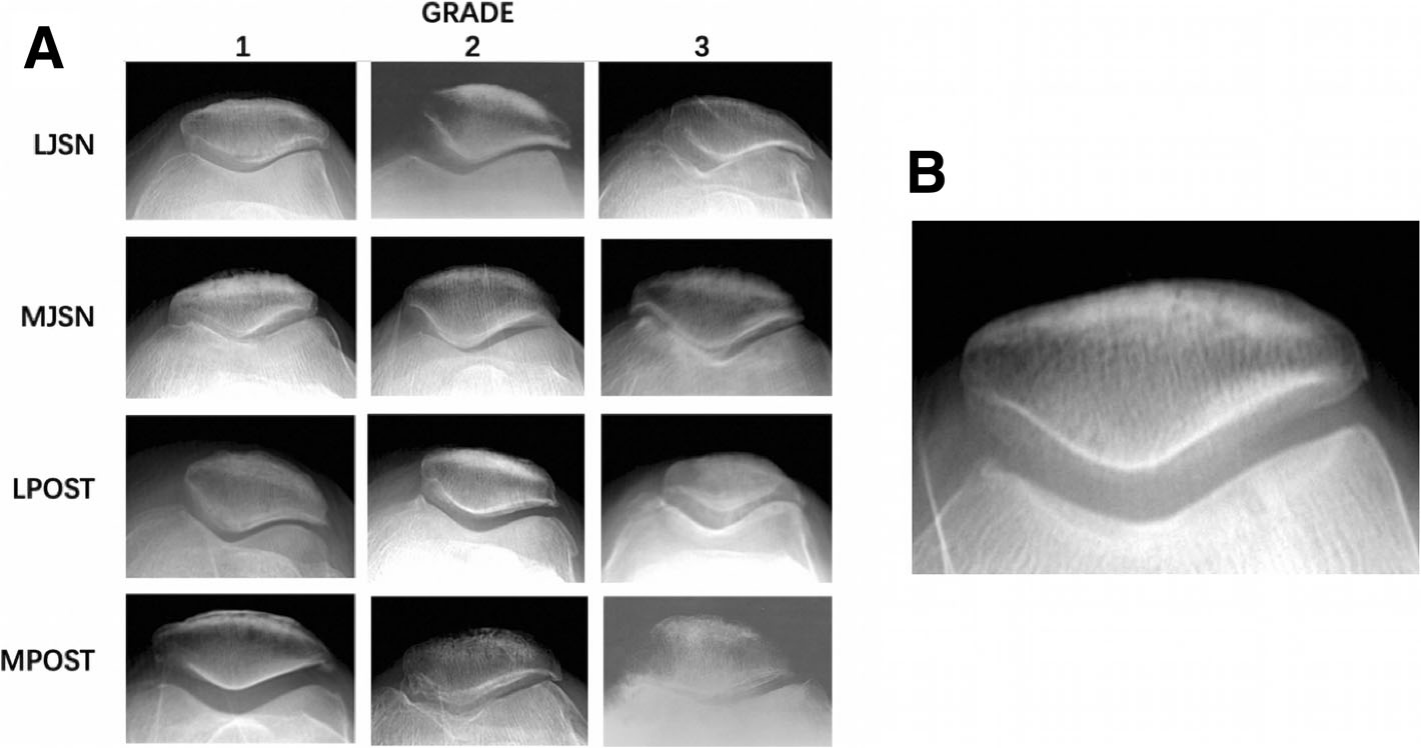
The normal radiograph (**b**, grade 0) is contrasted to grade1, grade2, and grade 3 abnormal radiographic features (**a**) on the skyline view of the patellofemoral joint Abbreviations. LJSN: lateral joint space narrowing; MJSN: medial joint space narrowing; LPOST: lateral patellofemoral osteophyte; MPOST: medial patellofemoral osteophyte.

The imaging data in 2014 were read by two experienced researchers (one musculoskeletal radiologist [QZ] and one osteologist [HL], both blinded to the data). Independently, inconsistent data were checked by another skilled researcher [XL]. The imaging data in 2017 were read by a researcher who was blinded to the data and completed intra-reader repeatability to kappa (> 0.8). For each batch of BJS study films (*n* = 100), 20 films from the 2014 were added to test inter-reader repeatability. In addition, 20 previously read PF radiographs from 2017 study were fed back to test intra-reader repeatability. The diagnostic disease severity grade atlas of PF radiographic features on the skyline view is shown in Fig. 1.

### Statistical analysis

Statistical analysis was done using SPSS (IBM, 25.0, CA, USA). The basic characteristics and clinical symptoms of the population were described by narrative statistics; independent-sample t-tests were performed to test the significance between the incident RPFOA patients and the whole population; and Kruskal-Wallis H tests were performed to compare results between the progress of lateral patellofemoral osteophyte (LPOST), medial patellofemoral osteophyte (MPOST), lateral joint space narrowing (LJSN), and medial joint space narrowing (MJSN). The results of analysis were presented as odds ratio (OR) with 95% confidence interval (CI), and a *p*-value < 0.05 was considered significant.

## Results

### Descriptive characteristics

A total of 1295 subjects were recruited at the baseline in 2014, and 967 (74.7%) were followed in 2017. The BJS study population was predominantly female (63.3%). The mean age was 60.3 years (SD = 6.60). The mean BMI was 26.4 (SD = 3.63). The mean knee ROM was 127.8 degrees (SD = 9.24). A total of 117 subjects had knee pain symptoms (12.1%) (Table 1).

**Table 1:** Descriptive characteristics comparation between incident RPFOA patients and total population

	Incident RPFOA Patients (119)	Total Population (967)	*P* value
Gender			< 0.01
Male, n (%)	36 (30.3)	345 (35.7)	
Female, n (%)	83 (69.7)	622 (63.3)	
Age, years			0.041
Mean (SD)	61.6 (6.44)	60.3 (6.60)	
BMI			0.032
Mean (SD)	27.2 (3.99)	26.4 (3.63)	
ROM			0.052
Mean (SD)	126.2 (8.10)	127.8 (9.24)	
Knee pain, n (%)	21 (17.6)	117 (12.1)	< 0.01

Abbreviations. *RPFOA* radiographic patellofemoral osteoarthritis, *BMI* body mass index, *ROM* range of motion

Among 1537 knee joint images without RPFOA at baseline, 139 cases (knee joint level) were diagnosed as incident RPFOA cases in 3 years. The incident RPFOA population was predominantly female (69.7%). The mean age was 61.6 years (SD = 6.44). The mean BMI was 27.2 (SD = 3.99). The mean ROM of knee joint was 126.2 degrees (SD = 8.1). A total of 21 incident RPFOA patients had knee pain symptoms (17.6%) (Table 1). Compared with the whole population, age (*P* = 0.041), BMI (*P* = 0.032), gender (male / female were valued 0/1, *p* < 0.01), and incidence of knee pain symptoms (*p* < 0.01) in incident RPFOA patients achieved significance, while ROM (*p* = 0.052) showed no significance.

### Imaging indicators measurement

Unweighted κ coefficients for inter-reader and intra-reader repeatability in 2017 were 0.82 and 0.85, respectively. Among 139 incident RPFOA radiographs, the progress of the imaging indicator grade was analyzed. The progress of marginal PF osteophyte was more pronounced than joint space narrowing, while the mean grade progress of LPSOT is 1.02, showed significance when compared to that of MPOST (0.49), LJSN (0.30), MJSN (0.06), and *p* < 0.01. The composition of each imaging indicator grade progress of incident RPFOA is shown in Table 2. The comparison *p*-values regarding the progress between the four indicator grades are shown in Table 3.

**Table 2:** Imaging indicator grade progress of incident RPFOA in 3 years

Progress of Imaging Indicator Grades							
	0	1		2		3	
LJSN, n (%)	108 (77.7)	0–1*	12 (8.6)	0–2*	8 (5.8)	None	
		1–2*	9 (6.5)	1–3*	2 (1.4)		
MJSN, n (%)	132 (95.0)	0–1*	6 (4.3)	0–2*	1 (0.7)	None	
LPOST, n (%)	35 (25.2)	0–1*	13 (9.4)	0–2*	36 (25.9)	0–3*	1 (0.7)
		1–2*	54 (38.8)				
MPOST, n(%)	89 (64.0)	0–1*	19 (13.7)	0–2*	18 (12.9)	None	
		1–2*	13 (9.4)				

*Indicates that the progress of each imaging indicator grade of the RPFOA radiographs increases from 0 to 1 (from 1 to 2, from 1 to 3, or from 0 to 3)

Abbreviations. *LJSN* lateral joint space narrowing; *MJSN* medial joint space narrowing; *LPOST* lateral patellofemoral osteophyte; *MPOST* medial patellofemoral osteophyte

**Table 3:** The p-values between the progress of imaging indicator grades of incident RPFOA

	MJSN (0.06)	LPOST (1.02)	MPOST (0.49)
LJSN (0.30)	0.01	< 0.01	0.114
MJSN (0.06)	n.s.	< 0.01	< 0.01
LPOST (1.02)	n.s.	n.s.	< 0.01

Abbreviations. *n.s* no significance; *LJSN* lateral joint space narrowing; *MJSN* medial joint space narrowing; *LPOST* lateral patellofemoral osteophyte; *MPOST* medial patellofemoral osteophyte

## Discussion

This is the first population-based, longitudinal, and prospective study demonstrating imaging features in the incident RPFOA population. Interestingly, we found that patella osteophyte progress faster than joint space narrowing. The progress of LPOST is significantly faster than the other three indicators, suggesting that PF joint lateral wear is the most common to be found in the incident RPFOA patients, and the formation of osteophyte is easier than that of joint space narrowing. PFOA is caused by the wear of patellar or trochlear groove cartilage, which is more common to be found in the lateral patellar region [12]. This indicates that the pressure on the lateral patella is greater than that on the central and medial patella. Baker et al. [9] found that quadriceps femoris weakness was more pronounced in patients with knee pain, and quadriceps femoris weakness was closely related to PFOA and tibiofemoral osteoarthritis (TFOA). Their study also showed that lateral compartment was more affected than medial compartment. When the knee buckles at 30 degrees, the abnormal pulley geometry reduces the lateral stability by 70%, while the relaxation of the medial oblique femoris reduces the lateral stability by 30% [13]. There are complex interactions among these anatomical structures. They have different effects on lateral stability of PF joint in knee flexion. Their long-term effects may cause imaging changes of the PF joint.

However, the relationship between the imaging features of RPFOA and clinical symptoms and signs is not as typical as that of radiological tibial femoral arthritis (RTFOA). In our study, we found that the ROM in knee joint did not show significant difference between the incident RPFOA patients and the whole population. Although the incidence of knee joint pain was significantly different between the two groups, the rate of knee pain in incident RPFOA patients (17.6%) was not as high as expected. A cohort study of 409 people by Camille Parsons et al. [14] demonstrated that clinical symptoms including knee bounce, patellar floating sign, knee tenderness and knee flexion pain were closely related to the increased risk of RTFOA, but only knee tenderness was associated with RPFOA. This is basically consistent with our findings, which may reflect that the contribution of imaging changes in isolated RPFOA are not as significant as the RTFOA in terms of the overall knee joint symptoms and physical signs. The symptoms of knee pain may be closely related to its overuse and injury history. Hip-muscle dysfunction, weakness of core muscle groups, excessive foot rotation, and patellar dislocation can also cause or aggravate knee pain symptoms [15]. Isolated RPFOA cannot be equated with knee joint symptoms.

Based on the results of our study, among all the incident RPFOA population, the proportion of women is higher than men, and the BMI of the incident RPFOA population is higher than that of the normal population. Besier et al. [16] found that quadriceps femoris muscle strength, especially medial muscle strength, plays an important role in maintaining patellar stability. In addition, muscle strength plays an important role in controlling the relative contact area between patella and femur under weight-bearing conditions. This may be the reason for the higher proportion of women. The study of Clark et al. [17] showed that the relative contact area between patella and femur increased by 38% when the muscle strength of medial femoral muscle increased from 0 N to 100 N at 30-degree knee flexion. This demonstrates that greater medial femoral muscle strength can lead to the increasement of the PF contact area; thereby the stability of the PF joint can be increased and the wear of the PF joint can be reduced. The pressure of the knee compartment is higher in people with higher BMI, which makes it more likely to form imaging progresses of the PF joint.

The diagnosis of RPFOA is usually interpreted by skyline view (45-degree) of the patellofemoral (PF) joints [18]. The skyline view is more accurate than lateral radiographs in reading joint space narrowing and patella osteophyte [19, 20]. With this information in mind, all the RPFOA diagnoses in the BJS study were based on skyline view for higher accuracy. The view chosen to define RPFOA may be critical [21]. A limitation of our study was that there was no gold standard for RPFOA diagnosis in international guidelines. Also, studies often have incomplete views of the knee joint. When the views are extended to encompass all aspects of the knee joint, the prevalence of radiographic changes of osteoarthritis may be higher. Because of the needs of our study, we redefined RPFOA based on definitions in previous articles [8, 9] by OARSI and its atlas. We consider that this definition to be feasible and to have a quantitative standard in clinical practice.

There are still other limitations of our study. Firstly, the population measurement in this study was localized in the Beijing area, which could have possibly influenced the precision of the results. Secondly, due to the relatively small population in Beijing engaged in heavy manual labor, the number of incident RPFOA cases may have been underestimated. Third, the follow-up duration of our study was 3 years, which was relatively shorter than in other studies. Given that the development of RPFOA is unlikely to occur in a short time, a longer period of observation is required to validate our findings. Fourth, radiographs cannot detect minor osteoarthritis changes when compared with magnetic resonance imaging (MRI) studies. We chose radiographs because they were easier to operate and relatively inexpensive. In addition, this study did not further classify isolated RPFOA and mixed (RPFOA + RTFOA) knee osteoarthritis, which are difficult to gauge as accurate reflections of the imaging features of RPFOA in the general population.

## Conclusions

RPFOA is often accompanied by knee pain and other symptoms. Although it is not as closely associated with knee joint symptoms as RTFOA, it still affects people’s quality of life to a great extent. Women, older people, and people with high BMI and knee pain compose the incident RPFOA population in greater proportion than in the overall population. In this study, the imaging features of incident RPFOA are briefly described: PF osteophyte is more pronounced than PF joint space narrowing, and LPOST is the most common imaging manifestation of RPFOA, with the fastest progress in severity grade. We hope that this study will enable people to have a clearer understanding of PFOA and to make efforts toward greater public health in China.

## Data Availability

Data will be available upon request by the first author YQ.

## Abbreviations

BMI: Body mass index
LJSN: Lateral joint space narrowing
LPOST: Lateral patellofemoral osteophyte
MJSN: Medial joint space narrowing
MPOST: Medial patellofemoral osteophyte
MRI: Magnetic resonance imaging
OA: Osteoarthritis
OARSI: Osteoarthritis Research Society International
PF: Patellofemoral
PFOA: Patellofemoral osteoarthritis
ROM: Range of motion
RPFOA: Radiographic patellofemoral osteoarthritis
RTFOA: Radiographic tibiofemoral osteoarthritis
TF: Tibiofemoral
